# Surgical treatment of giant right ventricular fibroma for a newborn: A case report

**DOI:** 10.3389/fcvm.2022.908287

**Published:** 2022-07-19

**Authors:** Yuhang Liu, Ning Wang, Ping Wen

**Affiliations:** Dalian Women and Children's Medical Center (Group), Dalian, China

**Keywords:** cardiac tumor, fibroma, case report, histology, newborn

## Abstract

This report describes the surgical treatment of giant right ventricular fibroma in a newborn. Cardiac uhrasonography and CT showed a large mass in the right ventricle wall, which narrowed the right ventricular inflow tract. The newborn patient gradually developed symptoms such as shortness of breath, oliguria, and pericardial effusion. We performed tumor excision, but due to severe damage to the right ventricular wall and right heart failure, the patient relied on cardiopulmonary bypass. Then, we immediately restored the opening of the ductus arteriosus, enlarged the foramen ovale, and used various vasoactive drugs to ensure the smooth resuscitation of the patient. This is a kind of operation for the youngest patients. The perioperative treatment experience indicated the feasibility of excision of giant right ventricular fibroma for newborn patients.

## Introduction

Primary heart tumor in children is quite rare. As reported, the incidence is about 0.03–0.32%, about 25% of the cases are fibroma, followed by benign heart tumor (1, 2). Cardiac fibromas are generally solitary and large at the free end of the left ventricle, while rare at the right ventricle or atrium. They may be aggressive tumors with high mortality and generally show progressive growth, with rare spontaneous regression. Therefore, in the case of any tumor-induced symptom or expected life-threatening complication, excision is the preferred treatment method. In this case, the operation was performed for a newborn (birth weight: 3.2 Kg) 6 days after birth for improving hemodynamic disorder due to giant right ventricular fibroma.

## Case summary

**Figure 1:**
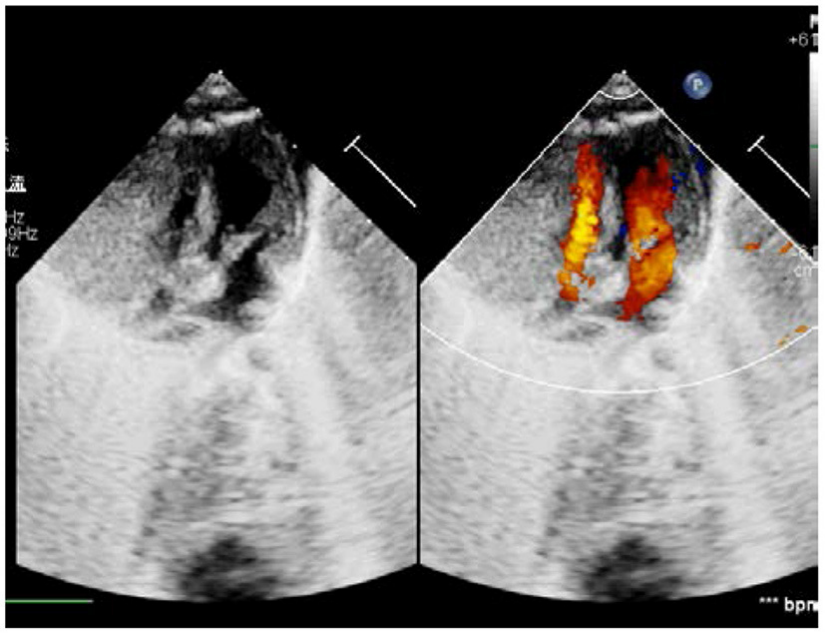
Ultrasonic Cardiogram: Neoplastic mass on the right ventricular wall and constricted right cardiac cavity.

**Figure 2:**
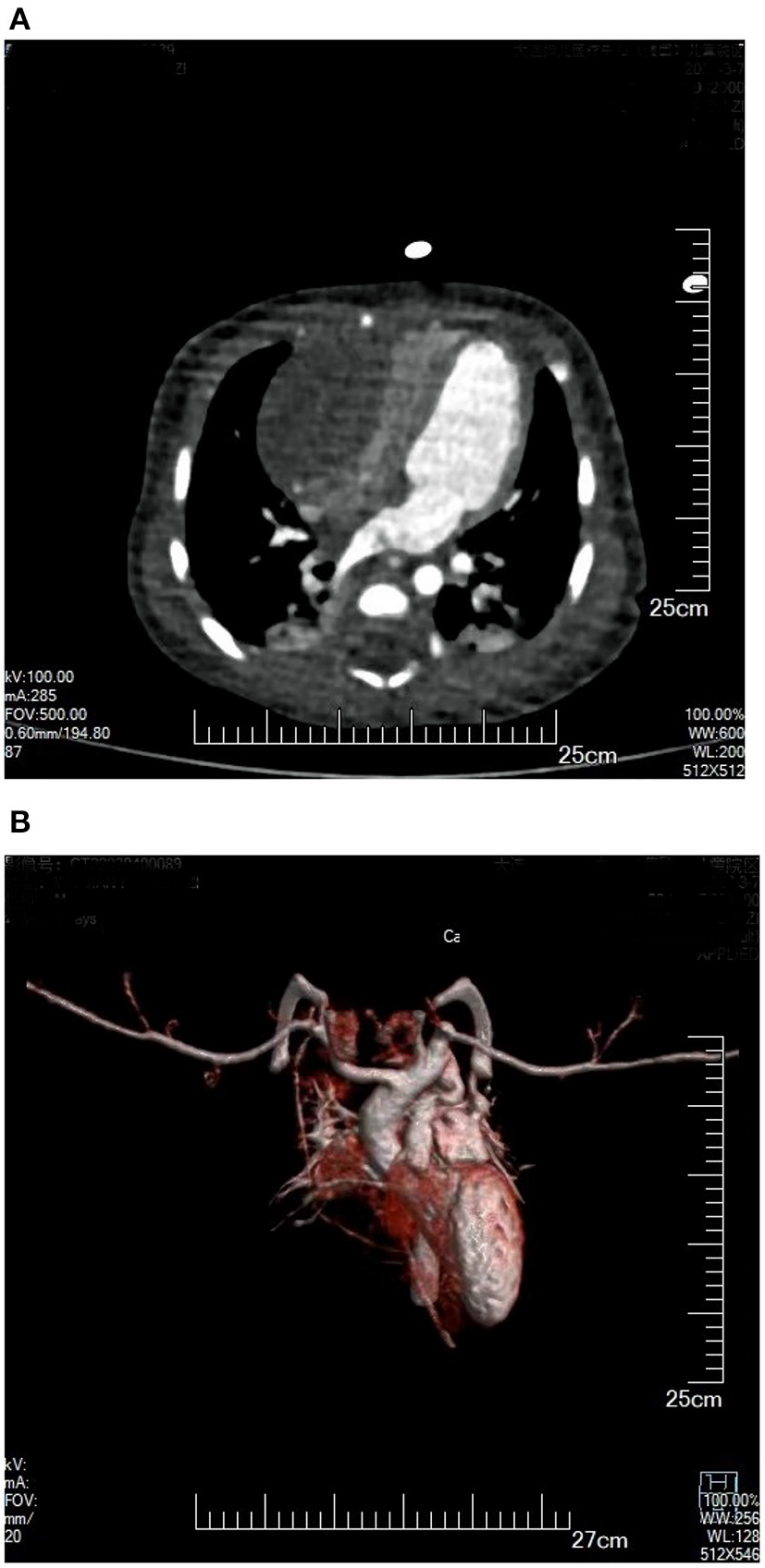
**(A)** Plain CT scan: Enormous space-occupying mass on a right ventricular wall, with compressed right ventricle cavity. **(B)** 3D reconstruction of cardiac blood flow: The right ventricular inflow tract was compressed by tumor, without obvious blood flow filling.

**Figure 3:**
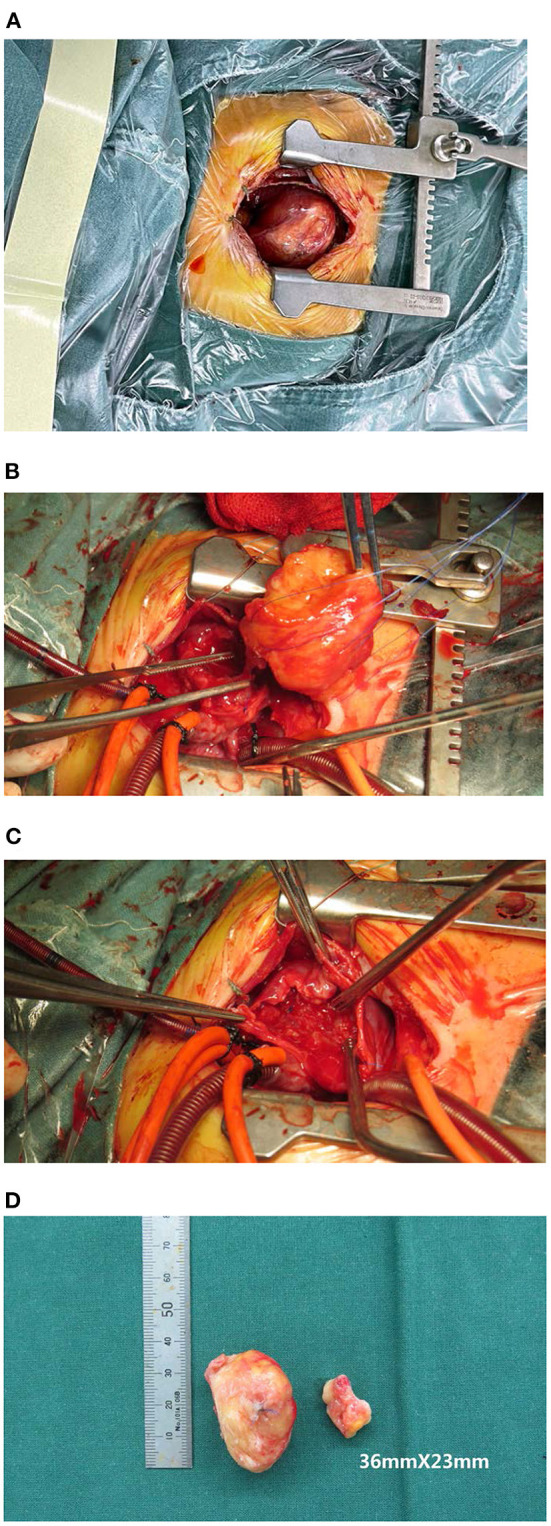
**(A)** Tumor appearance before an operation; **(B)** Removal of a tumor; **(C)** During operation, right ventricular wall wound after tumor removal; **(D)** Tumor specimen appearance.

**Figure 4:**
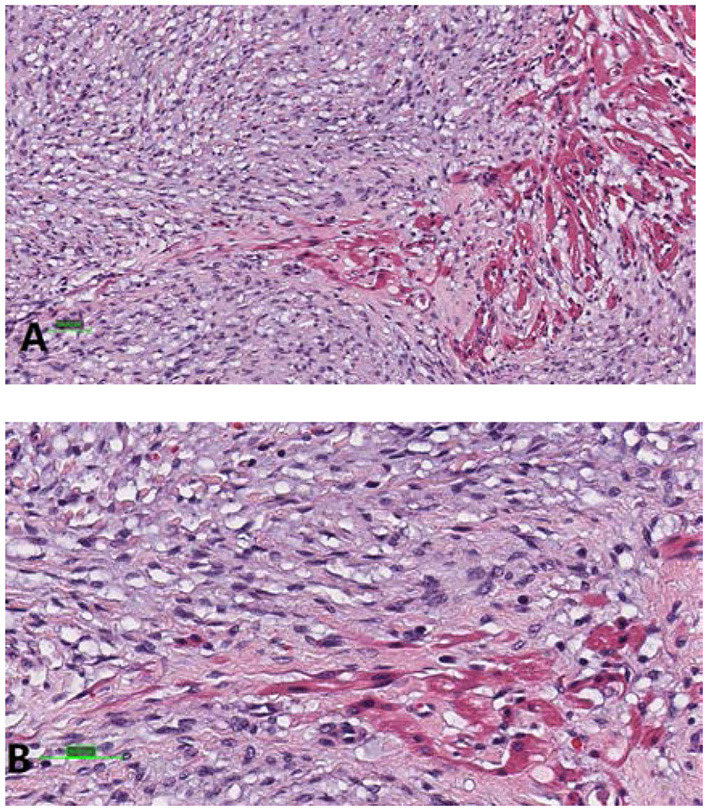
Examination under a light microscope **(A);** Magnification: 20x, and **(B)** 40x (hematoxylin and eosin staining).

The study protocol was approved by the Dalian Women and Children's Medical Center (Group) Institutional Ethics Committee. The legal guardian signed informed consent for the operation and clinical record review. The patient is a test-tube baby, which means that the baby was a result of *in-vitro* fertilization. Fetal cardiac uhrasonography showed solid mass occupying the right ventricular wall and pericardial effusion; therefore, the patient was transferred to Cardiac care unit (CCU) after birth, early vital signs were stable and physical examination showed no obvious abnormalities. Cardiac uhrasonography: A hypoechoic mass of about 36 mm × 23 mm on the right ventricular wall, narrowing of the right ventricular inflow tract (Figure 1), a small amount of pericardial effusion, patent foramen ovale (2 mm), and patent ductus arteriosus (2 mm). Cardiac CT angiography (CTA): An elliptical low-density shadow of 35 mm × 23 mm on the right ventricular wall, with the right ventricle compressed (Figures 2A,B). The patient gradually had shortness of breath, rapid heart rate and oliguria, and cardiac echocardiography indicated increased pericardial effusion during observation. Tumor excision and ligation of ductus arteriosus were performed on Day 6 after birth. During the operation, the tumor was found to be yellow and hard, with a clear boundary with the myocardial tissue. Part of the myocardial tissue was enlarged along the boundary between the tumor and the myocardial tissue, and the marginal myocardial tissue was cut from multiple locations for pathological examination to ensure complete tumor resection, the fibroma was completely removed (Figures 3A–C), and the right ventricular wall wound was directly sutured. After the first cardiopulmonary bypass, the patient had severe hypotension and hypoxemia, making the condition unsustainable. The cardiopulmonary bypass was arranged again; given severe damage to the right ventricular wall due to tumor excision, and right heart failure, the ductus arteriosus was opened, the foramen ovale was enlarged, and various vascular drugs such as dopamine, adrenaline and prostaglandin were given; the patient was barely out of cardiopulmonary bypass, and transferred to the ICU with delayed sternal closure. The patient's vital signs were gradually stable, the chest was closed on Day 3, the respirator was removed on Day 6, and the patient was discharged on Day 14 after an operation. At present, the heart function has returned to normal, with a satisfactory prognosis. Pathologic results confirmed the presence of fibroma (Figures 4A,B).

The patient underwent TTE, CT, and electrocardiography at the outpatient clinic every 3 months after surgery. So far the recovery has been good and the tumor has not returned.

## Discussion

Although cardiac fibroma is histopathologically benign, it may still be an aggressive tumor, with high mortality (approximately 20–33%) among primary cardiac tumors. Cardiac fibroma generally has no clear boundary with cardiac muscle tissues, therefore, there would be high rates of ventricular arrhythmia (64–89%) and sudden cardiac death (10–30%) (3, 4). In addition, cardiac fibroma is generally progressive, with rare natural regression. Therefore, in the case of any tumor-induced symptom or expected life-threatening complication, excision is the preferred treatment method. In this case, we expected to perform tumor excision when the patient was older, but on Day 6 after birth, the follow-up in ICU found clinical symptoms; we had to operate during the neonatal period.

The excision of a large tumor is likely to cause insufficient myocardial quality, thus leading to severe impairment of cardiac function. A literature review indicated that most deaths were caused by the failure in withdrawal of cardiopulmonary bypass and heart failure. Some researchers adopted extracorporeal membrane oxygenation (ECMO), but the long-term use resulted in a high rate of serious complications; while some performed subtotal excision of a tumor to avoid damage to the basic structure, but there was a high recurrence rate. In this case, the patient had severe right heart failure after the operation; we enlarged the foramen ovale, to make full right-to-left shunt and ensure smooth blood circulation; maintained the ductus arteriosus open, to increase pulmonary blood supply, which ensured basic oxygen supply, made the patient successfully live without cardiopulmonary bypass after an operation, and recover without ECMO.

## Conclusion

In conclusion, we performed excision of giant right ventricular fibroma for a newborn who had severe right heart failure after an operation but discharged after treatment. This is a kind of operation for the youngest patients worldwide. A large cardiac fibroma may lead to early blood flow obstruction or arrhythmias, which should be excised as soon as possible. It is possible to operate during the neonatal period.

## Data availability statement

The original contributions presented in the study are included in the article/supplementary material, further inquiries can be directed to the corresponding author/s.

## Ethics statement

This study protocol was approved by the Dalian Women and Children's Medical Center (Group) Institutional Ethics Committee. Written informed consent was obtained from the patient's legal guardian/next of kin for the publication of this case report. Written informed consent was obtained from the patient's legal guardian/next of kin for the publication of any potentially identifiable images or data included in this article.

## Author contributions

PW designed the study and performed the experiments. YL and NW performed the experiments, analyzed the data, and wrote the manuscript. PW was major contributor in writing the manuscript. All authors contributed to the article and approved the submitted version.

## Conflict of interest

The authors declare that the research was conducted in the absence of any commercial or financial relationships that could be construed as a potential conflict of interest.

## Publisher's note

All claims expressed in this article are solely those of the authors and do not necessarily represent those of their affiliated organizations, or those of the publisher, the editors and the reviewers. Any product that may be evaluated in this article, or claim that may be made by its manufacturer, is not guaranteed or endorsed by the publisher.
